# Sonic-induced cellular vibrations unzip intertwined anther cone trichomes to trigger floral self-pollination and boost tomato fruit size

**DOI:** 10.1093/hr/uhaf053

**Published:** 2025-02-19

**Authors:** Sidra Anwar, Happy, Angus Dingley, Thailammai Vinoth, Weiguang Liang, Brian M Sindel, Laurel George, Chun H Wang, Christopher I Cazzonelli

**Affiliations:** Hawkesbury Institute for the Environment, Western Sydney University, Locked Bag 1797, Penrith, NSW 2751, Australia; Agriculture and Food Research Unit, CSIRO, Clunies Ross Street, Acton, ACT 2601, Australia; Hawkesbury Institute for the Environment, Western Sydney University, Locked Bag 1797, Penrith, NSW 2751, Australia; Hawkesbury Institute for the Environment, Western Sydney University, Locked Bag 1797, Penrith, NSW 2751, Australia; Department of Agronomy and Soil Science, School of Environmental and Rural Science, University of New England, Armidale, NSW 2351, Australia; School of Mechanical and Manufacturing Engineering, University of New South Wales, Sydney, NSW 2052, Australia; Hawkesbury Institute for the Environment, Western Sydney University, Locked Bag 1797, Penrith, NSW 2751, Australia; Department of Agronomy and Soil Science, School of Environmental and Rural Science, University of New England, Armidale, NSW 2351, Australia; Hawkesbury Institute for the Environment, Western Sydney University, Locked Bag 1797, Penrith, NSW 2751, Australia; School of Mechanical and Manufacturing Engineering, University of New South Wales, Sydney, NSW 2052, Australia; Hawkesbury Institute for the Environment, Western Sydney University, Locked Bag 1797, Penrith, NSW 2751, Australia

## Abstract

Artificial tomato pollination methods rely on cellular vibrations from air displacement, electric vibration wands and trellis tapping, which have potential to spread pathogens. Bioacoustic frequencies emitted from buzzing bees to ultrasonication can vibrate plant cells without physical contact. The effects of frequency-dependent sonication on the poricidal anther cone sheath, self-pollination, seed set, and fruit size remain unclear. We engineered devices to investigate the frequency-dependent power-law behaviour of floral living cells from greenhouse-grown tomato varieties—contrasting contact-induced oscillations from a vibrating wand and mechanical shaker arm with precision noncontact sonication emitted by a subwoofer speaker. The velocity and acceleration of vibrating flowers and impact on poricidal anther cone sheath cellular structures, self-pollination, and fruit development were assessed. Sonic frequencies ranging from 50 to 10 000 Hz increased pollination, fruit size, weight, and seed set in Sweetelle, Endeavour, Paulanca, and Managua commercial varieties. Scanning electron microscopy revealed sonication separated the intertwined trichomes and unzipped their meshed network that locks the lobes of the anther cone sheath together thereby releasing pollen grains. Near ultra-sonic frequencies boosted fruit size, whereas seed set remained constant thereby challenging the floral cell power-law rheological characteristics under different frequency scales. Tomato flowers displayed a low power-law cell behaviour to frequency-dependent sonication enabling its effectiveness as a precision noncontact technology to boost pollination and tomato fruit size without a substrate-borne component.

## Introduction

The global area cultivated for pollinator-dependent crops has rapidly increased relative to pollinator-independent crop regions [1]. Agroecosystem activity has contributed to a loss in biodiversity and impacted upon natural pollinators [2]. The European honeybee (*Apis mellifera* L.) and northern hemisphere bumblebee (*Bombus* spp*.*) are very effective crop pollinators and their services necessitates periodic relocation of hives to dependent crops [3, 4]. In more recent times, the expansion of horticultural crops like almonds (*Prunus dulcis*), blueberries (*Vaccinium* spp.), and tomatoes (*Solanum lycopersicum*) requires increased pollinator movements [5, 6]. Biosecurity laws in certain countries like Australia prohibit the importation of non-native bumblebee pollinators due to a devastating threat that they could cause to ecological habitats [7]. World-wide pollination services have been threatened by the spread of parasitic mite pests such as *Varroa destructor*, which attack endemic as well as commercial honeybees and broods [8]. Therefore, managing natural pollinators can pose a multifaceted challenge, particularly within protected cropping facilities that are specifically designed to ensure superior quality and consistency of fresh market produce through precise environmental control [7, 9, 10]. As a result, protected cropping facilities incur a significant labour cost associated with manual-pollination services required for producing fresh-market tomatoes. Precision artificial pollination services have potential to add value by sustaining ecological biodiversity and enhancing horticultural crop production [11].

The demand for automating artificial pollination has prompted the development of electronic wands capable of inducing cellular vibrations, as well as the utilization of air-displacing drones and air-jets to mechanically stimulate flower movement. These methods replicate the natural interactions between plants and insects during foraging, facilitating the dispersion of pollen [11–13]. Artificial cellular vibration devices significantly increase self-pollination compared with a no-pollination control. One caveat is that outcrossing pollen from one flower to the stigma of another on the same plant (geitonogamy; a kind of self-pollination) or to the flowers on a different plant (xenogamy) produces the best, most fit, fruit and seeds. Hence it is not uncommon that artificial-induced self-pollination, such as in the case of cleistogamy, a phenomenon where plants self-pollinate via a single flower, elicits inferior outcomes when compared with bee pollinators that have various capacities to generate mechanical and buzz-induced vibrations capable of ensuring outcrossing pollination [13–15]. Buzz pollination encompasses the evolutionary convergence of specialized floral morphologies and pollinator behaviours by which bees use both mechanical and perhaps sonic means to vibrate flowers and release pollen [16, 17]. Bees, especially bumblebees (*Bombus* spp.), produce greater thoracic vibrations than other pollinators, generating capacity to move floral structures as well as induce sonic vibrations ranging from 100 to 400 Hz depending upon the bee–flower coupling [16, 18, 19]. From the plant perspective, how the frequency of noncontact cellular vibrations from sonics interplays with floral characteristics to modulate pollen release and contribute to buzz pollination remains unclear.

The mechanisms by which pollinators contribute to reproduction vary among plant species. While some plants rely on the simple translocation of available pollen to receptive stigmas, others necessitate specific stimuli to activate the male physiology and make pollen available [20, 21]. Dioecious species need pollen vectors for their gametes to unify, whereas monoecious demand a similar but different role from the pollinator [22, 23]. Tomato plants produce self-fertile hermaphroditic flowers and have a unique reproductive strategy that requires specific stimulation of the anthers to release pollen [24]. Tomato flowers develop an anther cone sheath of tissue layered around five anthers fused together that forms an open hollow tube-like morphological structure to the stigma and style. The formation of a stigma-enclosing anther cone sheath leads to a type of self-pollination referred to as cleistogamy, which provides a genetic stabilization of desired agronomic traits as well as a higher fruiting rate [25]. Intertwined (or interlocking) trichomes comparable to the teeth of a jigsaw puzzle provide a structural connection to unite neighbouring anthers and secure pollen within the closed anther cone sheath thereby promoting cleistogamy [25–27]. Successful pollen extraction can require vibratory forces produced by physical contact with a bee since its mass collectively gives rise to an axial-bending vibration mode when combined with the dynamical properties of the poricidal anther and supporting filament [19]. This restriction mechanism is hypothesized to meter pollen expulsion according to the abundance and transfer efficiency of pollinators resulting in maximum pollen utilization [24]. The arrangement of poricidal anthers found in tomato flowers is distinguished between insects on the proxy of their vibration capacities [28]. The efficacy of low-level mechanical vibrational frequencies of flowers generated by artificial devices are like those produced by bees and can adequately pollinate glasshouse-grown tomatoes [11]. Some studies indicated that pollen expulsion could be correlated to vibration amplitude rather than frequency in some species having poricidal anthers [29–31]. Fruit size, seed set, and postharvest quality correlates with the extent of successful pollination and fertilization [32]. It remains unclear as to whether higher frequencies of vibrational stimulation can propagate more energy to achieve superior self-pollination outcomes for tomato flowers.

Energy transmission from the pollinator to the floral structures involves physical and acoustic mechanisms [33]. Pollinator taxa display varying sound generating capabilities [34]. The role of the acoustic component has previously been understated as a by-product of mechanical stimulation, yet it can trigger energy transmission to the pollen grains [18]. The precise mechanism by which vibrational energy exerts inertia upon individual pollen grains, allowing their escape from the anther locule, remains unclear. A combination of centrifugal, kinetic, and electrostatic forces is thought to be responsible [35]. Vibrations vary according to differentials in amplitudes of displacement, acceleration, and velocity [36]. Hang and co-authors put forward a theory that described the response of living cells to stimuli of different frequencies, positing that the behaviour of living cells can be accurately described by a frequency-dependent power-law. In the low-frequency range (1–100 Hz), living cells exhibit a weak dependence on frequency, meaning their response to the stimulus changes within a specific lower frequency range. At high frequencies (100–10 000 Hz), however, living cells exhibit a tendency towards a constant response, as they become less sensitive to the changes in frequency [37]. Plants have specific natural resonant frequencies, at which most of the vibration energy is concentrated when subjected to external stimulations. The natural frequencies depend on the plant's physical properties (e.g. mass, stiffness, moisture content) such as those influenced by turgor pressure [38]. Vibrations at natural frequencies can more efficiently propagate through tissues than other nonresonant frequencies [39], therefore nonresonant frequencies normally induce lower acceleration and are hence less effective for pollen expulsion [30]. The force–frequency relationship, duration, and repetition of cellular vibration events can lead to transient and/or long-lasting changes in plant growth, yield, and stress acclimation responses [40]. What remains unknown is whether the cellular response to contact and noncontact vibrational stimuli is dependent upon the resonant emission frequency and if the frequency-dependent power-law theory applies when using a broad range of sonication frequencies to induce floral self-pollination.

**Fig. 1:**
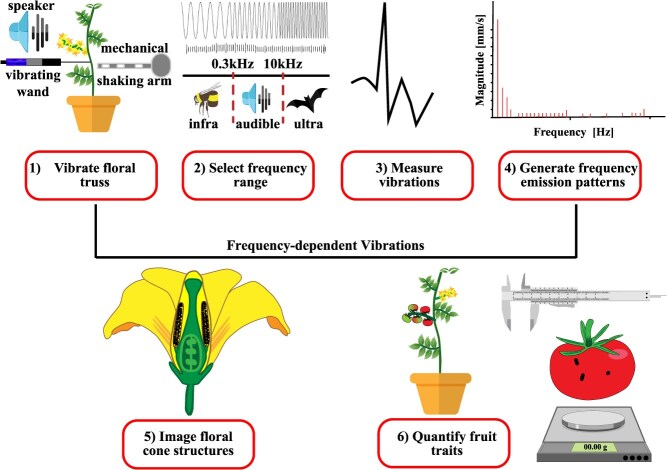
**Experimental approach towards engineering precision noncontact sonication technology to boost floral vibrations, self-pollination and tomato fruit development.** (1) Vibrate the floral truss. Vibrational stimulation of flowers on a tomato truss was performed using a subwoofer speaker, electric vibrating wand, and mechanical shaking arm. (2) Select the frequency range. Sonication frequencies exerted by the speaker (50 to 10 000 Hz) ranged from a = buzzing bee to ultrasonication by a bat. The vibrating wand (40 Hz) and aluminium shaking arm (40 to 300 Hz) were optimized to operate between infra to audible sonication ranges. (3) Measure vibrations. Floral vibrational frequencies and amplitude were measured using a laser vibrometer. (4) Generate frequency emission patterns. Floral acceleration was determined for all vibrational stimulation devices that promote cell movement. (5) Image floral structures. The poricidal cone, trichomes, and anthers of the tomato flower were imaged using scanning electron microscopy to understand the mechanism by which cellular vibrations promote pollen release. (6) Quantify fruit traits. The success of self-pollination was scored by measuring fruit weight, size, and seed set.

The objective of this study was to establish a comprehensive understanding of the influence of floral velocity and amplitude, induced by a diverse range of sonic frequencies ranging from 50 Hz to 10 kHz, on tomato pollination success and the underlying cellular structures involved in pollen release. We hypothesized that sonic vibration frequencies can significantly affect pollination outcomes in terms of fruit size, weight, and seed set and that higher sonication frequencies can outperform conventional mechanical contact pollination methods. It is worth noting that mechanical, contact-based excitation methods can only generate low frequency vibrations in flowers near the stems' natural frequencies. By contrast, high frequency vibrations can be induced directly into the flowers by using amplifier operated speakers. To this end, we employed two different methods to trigger floral vibration: an active subwoofer speaker to induce acoustic waves that directly induced vibration in the flowers and a mechanical shaker and electric vibrating wand that excited the stem. Four commercial tomato varieties were grown within a protected cropping environment and subjected to a bioacoustics experimental strategy. We quantified floral vibration responses and generated frequency emission patterns induced by three vibration sources. Vibrational frequency ranges were deployed to tomato flowers and a quantified strategy assessed fruit traits (Fig. 1). Our development of a noncontact sonication method offers the first step towards engineering a simple, scalable, and robotic method for automated pollination within the tomato cropping industry.

**Fig. 2:**
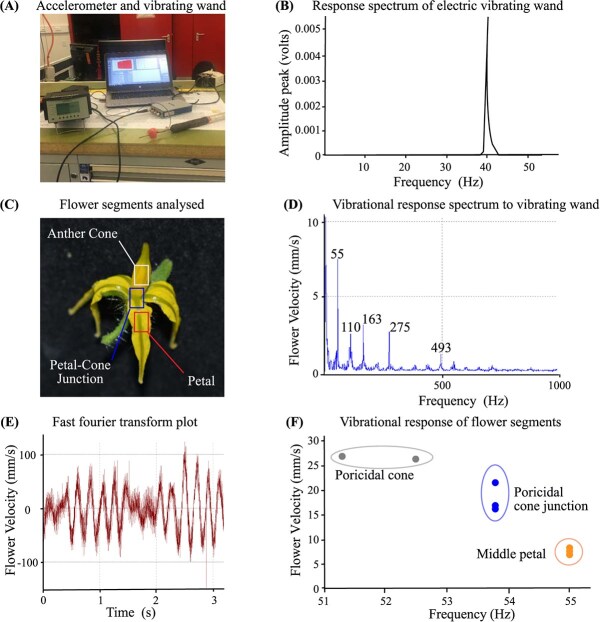
**Quantification of electric wand-induced floral vibrations using a laser vibrometer.** (A, B) Accelerometer used to measure the operating frequency (Hz) and amplitude (volts) response (dynamic body acceleration) emitted by an industry standard floral vibrating wand. The acceleration trace is representative of five experimental repeats. (C) Tomato flower showing the anther cone sheath (anther + stigma), middle petal, and petal/cone sheath junction used to measure vibrating wand-induced responses transferred from stimulating the main stem below the truss. (D) Vibrational response spectrum showing magnitude floral velocity (mm/s) relative to frequency (Hz) information when the stem was stimulated by the vibrating wand. (E) Fourier analysis transformation of the wand vibrational signal from the frequency domain to a representation in time (s) and velocity (mm/s). (F) Vibrational response of flower segments showing the floral velocity (mm/s) vibration relative to frequency (Hz) during mechanical stimulation of the main stem using an electric vibrating wand.

## Results

### Harmonic flower responses to stem vibrations are induced by an electric vibrating wand

An accelerometer, charge amplifier and data acquisition (DAQ) apparatus showed the frequency produced by the commercial electric vibrating wand peaked at 40 Hz (Fig. 2A and B). The velocities of three floral organs (anther cone sheath, petal, and petal-cone junction) during stem vibrations from an electrical wand were assessed using a laser vibrometer setup in the glasshouse (Fig. 2C). The flower vibrated at an average frequency of approximately 55 Hz, resulting in a vibration velocity of around 7.4 mm/s. Four additional harmonic frequencies (integer multiples of the fundamental frequency value caused by resonance of the vibrating flower) of 110, 163, 275, and 493 Hz were identified (Fig. 2D). Fourier transformation of the vibrational signal in time revealed spectral components indicative of a consistent flower velocity during a 3-second interval (Fig. 2E). The floral vibration frequency remained consistent across the three floral components, spanning from 51 to 55 Hz. Among them, the anther cone sheath exhibited the highest velocity (~26 mm/s), while the petal displayed the lowest velocity (~7 mm/s). The vibration velocity of the petal-floral axis junction (~20 mm/s), fell between the velocities observed for the anther cone sheath and petal (Fig. 2F). Therefore, mechanical vibrations via electrical wand to the plant stem below the truss induced the strongest and yet consistent vibrational response to the poricidal cone in a harmonic manner ranging from 55 to 493 Hz.

### Flower vibrational response spectrum to a mechanical shaking arm

**Fig. 3:**
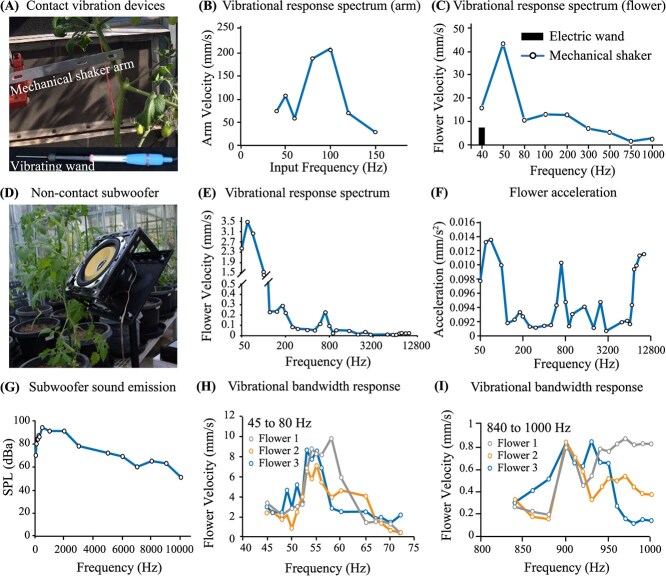
**Floral vibration response to frequency-dependent mechanical contact and noncontact sonic devices**. (A) Vibrational devices used to contact the plant stem below the truss and induce floral vibrations. Top image shows an aluminium arm from a mechanical shaker and lower image shows the electric vibrating wand. (B) Vibration velocity (mm/s) of mechanical shaker arm relative to input frequency (Hz). (C) Flower velocity (mm/s) response relative to frequency for both the vibrating wand and mechanical shaker arm quantified using a laser vibrometer. (D) Noncontact subwoofer speaker used to vibrate flowers via sonication. (E) Sonication-induced floral vibration response depicting vibration velocity (mm/s) relative to frequency. (F) Floral acceleration (μm/s^2^) relative to frequency generated by the subwoofer. (G) Sound pressure level (SPL) in weighted decibels (dBA) generated by the subwoofer across a wide range of frequency outputs (50 to 10 000 Hz). (H-I) Velocity (mm/s) of three flowers across narrow lower emission frequencies (45–80 Hz) and mid-range emission frequencies (840–1000 Hz) emitted by the subwoofer speaker. Experiments were repeated at least three times.

The mechanical arm output velocity was quantified relative to a sliding input frequency up to 150 Hz and two vibrational peaks were observed to range from 40 to 50 Hz and another stronger signal from 80–100 Hz, after which the arm velocity began to decline (Fig. 3A and B). The vibration response of tomato flowers to the mechanical shaking arm (40 to 1 kHz) were assessed in comparison to an electric vibrating wand and the floral vibrational velocity response at 40 Hz of mechanical arm movement was observed to be twice (~14 mm/s) that of the vibrating wand (~7 mm /s) (Fig. 3C). The vibration velocity of the flower was highest when the stem was vibrated at 50 Hz by the mechanical shaker arm (~40 mm /s), and rapidly declined to 80 Hz after which it gradually weakened unable to stimulate flower movement (Fig. 3C).

### Sonication induces floral vibrational bandwidth responses with higher acceleration

The floral response was measured during noncontact vibrations induced by a subwoofer speaker operating between 30 and 10 000 Hz (Fig. 3D). The highest floral velocity was observed at ~60 Hz (3.46 mm/s) and declined rapidly revealing two smaller peaks around 180 Hz (~0.29 mm/s) and 700 Hz (~0.23 mm/s) before stabilizing between 1 and 10 kHz at 0.047 to 0.018 mm/s, respectively (Fig. 3E). The acceleration of floral vibration was similar at 50, 700, and 10 000 Hz (0.13, 0.10 and 0.12 mm/s^2^, respectively) (Fig. 3F). Sonic vibrations at 1 000 Hz induced the highest sound pressure level (SPL) of ~94 dB and steadily decreased to ~50 dB at 10 000 Hz (Fig. 3G). The vibrational bandwidth response to a subwoofer treatment of three sequential flowers on a single truss was quantified using a laser vibrometer. Within a bandwidth range of 55 to 60 Hz, the three flowers showed a similar peak amplitude velocity of 8 mm/s (Fig. 3H). Within the frequency range of 840 to 1 000 Hz, the peak amplitude flower velocity of the three flowers reached its maximum at 880 Hz (~0.80 mm/s) (Fig. 3I). Therefore, the floral velocity was highest at lower sonic frequency bandwidth (50–60 Hz), and there were two other distinct peaks of high acceleration around 840 to 1000 Hz and 10 000 Hz.

### Sonication of Endeavour flowers induce effective self-pollination and a frequency-dependent boost in fruit size

**Fig. 4:**
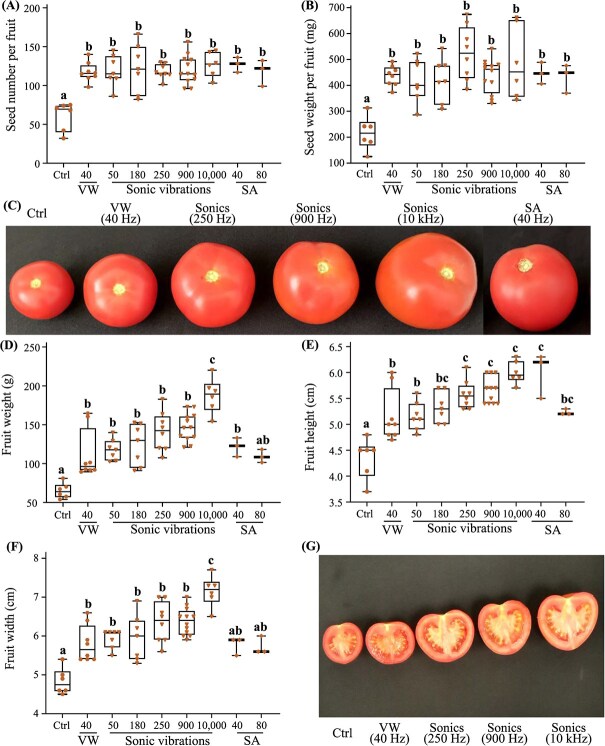
**Endeavour variety fruit set responses to mechanical contact and noncontact sonication-induced pollination.** The pollination efficacy of mature ripe fruits from untreated flowers (control; ctrl; n = 6), flowers mechanically stimulated using an electronic vibrating wand (VW; 40 Hz; n = 8) or a shaker arm (SA; 40 Hz and 80 Hz; n = 3), or flowers subject to sonication frequencies (50, 180, 250, 900, 10 000 Hz; n = 6 = 11) was assessed by counting (A) seed numbers and (B) seed weight (mg). (C) Representative mature ripe fruits shown. Postharvest tomato fruit qualities measured include (D) weight (g), (E) height (cm), and (F) width (cm). (G) Representative transverse sections of the tomato fruits treated with sonic vibrations (250, 900, 10 000 Hz) compared with the control (Ctrl) and electronic vibrating wand (VW). Experiments were conducted twice throughout the crop growth period, principally during a descending photoperiod from February to September. The box plots illustrate the median (central line marks the mid-point) and outer box lines as well as error bars mark the four quartile data marks. Individual data points represent fruits from multiple trusses and individual plants collected from two independent experiments (n = 4–11 fruits). Lettering denotes statistical analysis performed using a one-way ANOVA (*P* < 0.05).

Endeavour self-pollination efficiency to vibrational stimulation by an electric vibrating wand, mechanical shaker arm (40 and 80 Hz) and sonication (50, 180, 250, 900, and 10 000 Hz) was assessed by quantifying seed set (number of seeds/fruits, and seed weight/fruit), fruit size (fruit height and width), and weight relative to the untreated controls (Fig. 4). The number of seeds formed per tomato fruit reflects the pollination efficiency. All floral vibration frequencies, regardless of the treatment device, significantly enhanced seed number per fruit by ~1.9 to 2.1-fold (~94–110%) relative to the untreated controls (Fig. 4A). Floral vibration treatments substantially increased seed weight per fruit by 1.9 to 2.5-fold (94–143%) compared with the control (Fig. 4B). There were no significant differences in seed set among the three pollination treatments revealing that the lower frequencies (40–50 Hz) were equally effective as the higher frequencies (10 000 Hz) in triggering self-pollination (Fig. 4A and B).

**Fig. 5:**
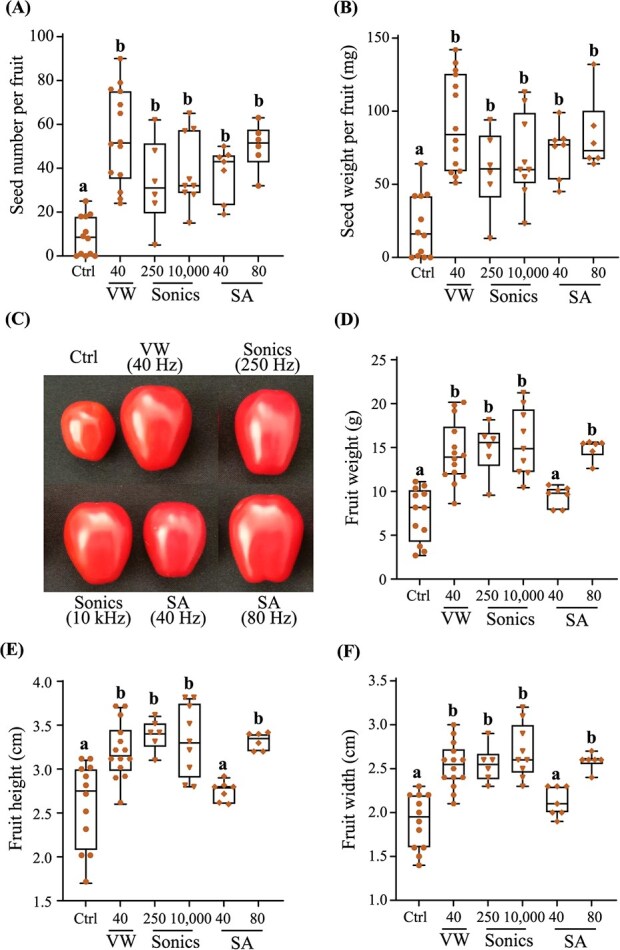
**Fruit and seed set responses to mechanical contact and noncontact sonication-induced pollination in Sweetelle variety.** Pollination efficacy of mature ripe fruits from untreated flowers (control; ctrl; n = 12), flowers mechanically stimulated using an electronic vibrating wand (VW; 40 Hz; n = 14) or a shaker arm (SA; 40 Hz and 80 Hz; n = 6–7), or flowers subject to sonication frequencies (250 Hz, and 10 000 Hz; n = 6–9) was assessed by counting (A) seed numbers and (B) seed weight (mg). (C) Representative mature ripe fruits shown. Postharvest tomato fruit qualities measured include (D) weight (g), (E) height (cm) and (F) width (cm). Experiments were conducted twice throughout the crop growth period, principally during a descending photoperiod from February to September. The box plots illustrate the median (central line marks the mid-point) and outer box lines as well as error bars mark the four quartile data marks. Individual data points represent fruits from multiple trusses and individual plants collected from two independent experiments (n = 6–14 fruits). Lettering denotes statistical analysis performed using a one-way ANOVA (*P* < 0.05).

**Fig. 6:**
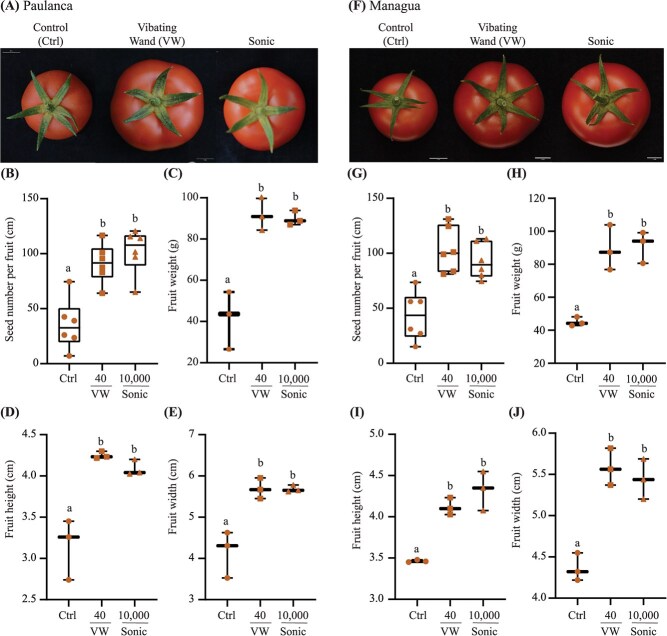
**Fruit size and seed set in Paulanca and Managua tomato varieties exposed to mechanical and sonication-induced pollination**. The electric vibrating wand (VW; 40 Hz) and sonication (Sonic; 10 000 Hz) were used to vibrate flowers and mature fruit traits from Paulanca (A–E) and Managua (F–J) were assessed relative to the untreated control (Ctrl). Panels (A) and (F) display representative top view image of mature ripe fruits. Pollination efficacy was assessed by counting seed numbers per fruit (B and G), fruit weight (C and H) height (D and I), and width (E and J). Experiments were conducted throughout a descending photoperiod in a cropping season from February to September in Australia. The box plots illustrate the median (central line marks the mid-point) and outer box lines as well as error bars mark the four quartile data marks. Individual data points represent the average number of fruits from two trusses (n > 12) collected from three individual plant replications (n = 3 fruits). Lettering denotes statistical analysis performed using a one-way ANOVA (*P* < 0.05).

Cellular vibrations enhanced the overall fruit size relative to the untreated control, yet the phenotypic variations depended on the specific vibrational treatment and frequency applied (Fig. 4C). Lower vibrational frequencies generated by the electric vibrating wand (40 Hz), and subwoofer (50 Hz) significantly enhanced fruit weight (72–82%), height (19%), and width (20–23%) (Fig. 4D–F). Mechanical arm vibrations at 40 Hz significantly increased fruit weight (87%) and height (38%) yet the width was only marginally larger compared with the control. At 80 Hz, the mechanical arm displayed significant increases in height (20%) and width (19%), while the overall weight showed only a minor upward trend compared to the control.(Fig. 4D–F). There was an overall linear trend indicating that fruit size increased with sonic frequency, with the distribution of fruit parameter plot ranges being notably higher between 250 and 10 000 Hz compared to lower frequencies. (Fig. 4C–F). Fruit height was significantly enhanced by sonic vibrations at 250 Hz (28%), 900 Hz (31%) and 10 000 Hz (38%) (Fig. 4E). The fruit width (31–48%) and weight (118–188%) were substantially higher at 10000 Hz (Fig. 4D and F). A cross-section of the tomato fruits revealed that sonication at higher frequencies enhanced the mesocarp thickness without altering seed set in comparison with the electric vibrating wand (Fig. 4G). Therefore, the highest sonic frequency of 10 000 Hz unexpectedly hyper-induced fruit height, width, weight, and mesocarp thickness revealing a robust frequency-dependent power-law cell behaviour observed in response to infra- to near ultra-sonic induced floral vibrations.

### Sweetelle flowers exhibit a weak power-law dependence towards sonic-induced self-pollination and fruit size

The pollination efficiency of cell vibrational frequencies induced by infra- (250 Hz) and near ultra-sonication (10 000 Hz), mechanical shaking arm (40, 80 Hz), and electric vibrating wand (40 Hz) were next evaluated using Sweetelle flowers (Fig. 5). The seed set and seed weight per fruit were similar across all treatments, while exhibiting a remarkable increase of 3.7 to 6-fold (265–493%) and 2.8 to 4.4-fold (182–333%), respectively, in comparison with the untreated control (Fig. 5A and B). The lower 40 Hz of mechanical shaking arm vibration did not change the fruit height, width, and weight. However, sonic frequencies (250 and 10 000 Hz), the 40-Hz vibrating wand, and 80 Hz of mechanical arm movement significantly enhanced fruit weight, height, and width, by 1.9 to 2.1-fold (94–109%), 1.2 to 1.3-fold (24–30%), and 1.3 to 1.4-fold (33–42%), respectively, compared with the control (Fig. 5C–F). Collectively, these findings illustrate that infra- and ultra-sonication can trigger self-pollination in Sweetelle and enhance fruit size effectively like that of the gold standard electric vibrating wand.

### Sonication of Paulanca and Managua flowers promotes effective self-pollination and enhances tomato fruit size.

Sonication (10 000 Hz) and the electric wand (40 Hz) were used to vibrate flowers from two commercial varieties, Paulanca and Managua, and the mature fruits appeared considerably larger compared to the untreated controls (Fig. 6A and F). Sonication and the vibrating wand significantly increased the seed number per fruit for both Paulanca (2.6 to 2.9-fold) and Managua (2.4 to 2.2-fold) compared to the controls (Fig. 6B and G). There were no significant differences in seed set among the two pollination treatments revealing that the lower frequencies (40) were equally effective as the higher frequencies (10 000 Hz) in triggering self-pollination. Sonic- and wand-induced floral vibrations similarly enhanced Paulanca fruit weight (121–116%), height (35–30%), and width (37–37%) (Fig. 6C–E). Likewise, a significant increase in Managua fruit weight (98–103%), height (19–25%), and width (28–25%) was observed relative to the control (Fig. 6H–J). These findings highlight the effectiveness of high-frequency noncontact sonication as an effective method to enhance pollination, seed set, and boost tomato fruit size in multiple varieties.

### Sonic vibrations separate intertwined trichomes between the lobes that connect the anther cone sheath

**Fig. 7:**
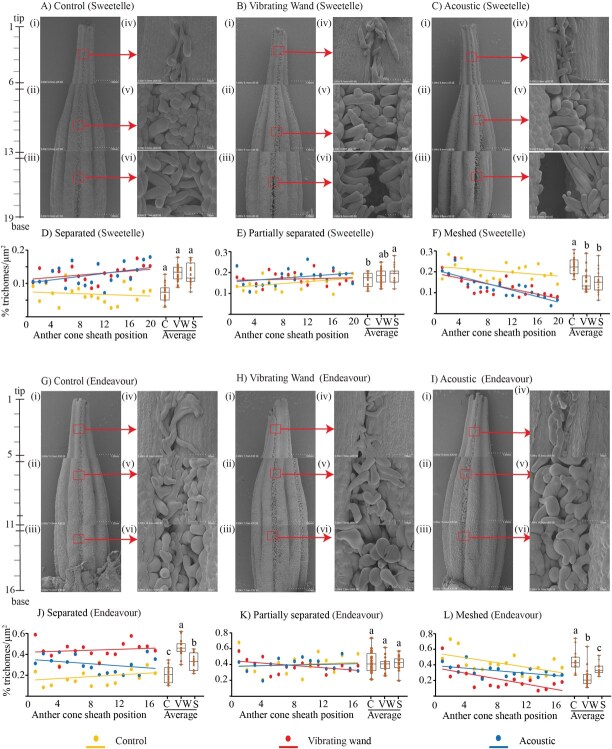
**Cellular imaging of sonication-induced changes to the anther cone sheath and trichome structures from Sweetelle and Endeavour flowers**. Flowers from Sweetelle (A–C) and Endeavour (G–I) plants were imaged after no treatment (A, G; control), treatment of the stem with a vibrating wand (B, H), or subject to 10 k Hz sonication (C, I). SEM images of the anther cone sheath (i–iii) or magnified (300x magnification) trichomes (iv–vi) displayed at the upper (i, iv), middle (ii, v) and lower (iii, vi) portions (35x magnification). The percentage of separated (D, J), partially separated (E, K) and meshed (F, L) trichomes relative to the total trichome number for each image were normalized per unit of total trichome area (n = 3 images per position/flower). The percentage trichomes types were scored along the tip to base of the anther junction between the two lobes and plotted relative to the anther cone sheath position. The bar graph shows the average percentage of trichome types for the entire another cone from tip to base (n = 19; Sweetelle, n = 16; Endeavour).

The mechanism by which sonic vibrations trigger pollen release was investigated by examining the anther cone sheath structure from untreated and mechanically vibrated flowers (40 Hz vibrating wand and 10 kHz sonication) from both Sweetelle and Endeavour varieties. Scanning electron microscope (SEM) revealed distinct morphological changes in trichome structures in Sweetelle and Endeavour flowers (Fig. 7). Untreated control poricidal cones exhibited uniform and tightly intertwined dorsal trichome structures between the lobes along the upper appendage (i, iv), middle (ii, v), and lower base (iii, vi) of the cone (Fig. 7A and G). Flower exposed to both the vibrating wand (Fig. 7B and H, i–iii) and sonication (Fig. 7C and I, i–iii) treatments displayed more separated (unentwined) trichomes in between the sheath lobes that appeared loosely packed with darker patches when compared with the untreated control (Fig. 7A and G, i–iii). There was an obvious visual separation in the middle and lower base anther cone sheath zones from Sweetelle flowers after treatment with the vibrating wand or sonication (Fig. 7B and C; v–vi). Endeavour flowers subjected to sonication appeared less compact compared to the untreated control, which did not show noticeable separation (Fig. 7G and I, v–vi), whereas, the vibrating wand caused subtle visual separation in the lower anther base zone (Fig. 7H, v–vi).

The dorsal surface between lobes of the anther cone sheath of Sweetelle flowers exhibited a significantly higher percentage of separated trichomes after treatment with the vibrating wand and sonication, compared to the control. The separation of trichomes gradually increased from the tip to the base along the cone sheath. (Fig. 7D). The percentage of partially separated trichomes was similar between the vibrating wand treatment and the control but was significantly higher in sonication-treated flowers along the length of the cone sheath. (Fig. 7E). The percentage of meshed trichomes declined gradually from tip to base and was significantly reduced in the vibrating wand and sonication treatments compared to the control. (Fig. 7F). Overall, a higher percentage of separated trichomes was observed, which inversely corresponded to a lower percentage of meshed trichomes following sonic vibration.

The vibrating wand and sonic treatments significantly increased the percentage number of separated trichomes along the Endeavour anther cone sheath from the upper tip to lower base when compared to the control (Fig. 7J). The vibrating wand was slightly more effective in separating the trichomes compared to sonicated flowers. The percentage of partially separated trichomes were similar for the vibrating wand, sonication, and control flowers along the upper stylar tip to lower anther cone sheath base (Fig. 7K). The percentage of meshed trichomes gradually declined along the upper stylar tip to lower cone base for all treatments, but the average was significantly lower in the wand and sonic treatments when compared to the control (Fig. 7L).

Overall, both Sweetelle and Endeavour anthers showed a significantly higher percentage number of separated trichomes that inversely correlated with a significant reduction in the number of meshed trichomes along the tip to base after exposing flowers to the vibrating wand and sonication treatments. Therefore, cellular vibration forces from sonication can effectively separate trichomes and disentangle their intertwined meshed network joining lobes within the anther cone sheath, thereby releasing pollen grains required for self-pollination.

## Discussion

Sound compression waves force plant cells and organelles to vibrate that triggers plant mechano-sensory receptors, signal transduction cascades, and the regulation of nuclear gene expression as well as chemical processes [42, 43]. We engineered new devices to investigate the frequency-dependent power-law behaviour of floral living cells from four commercial tomato varieties using contact-based mechanical (vibrating wand and/or mechanical shaker arm) and precision noncontact sonication (subwoofer speaker) as cell vibration-inducing methods (Fig. 1). Empirical studies suggest that mechanical characteristics of vibrations transmitted from insects or artificial mechanical stimulation by shaking can vibrate floral anther cells to release pollen and trigger pollination [30]. The floral vibrations produced by the vibrating wand, mechanical shaker arm and sonication are described with the same basic parameters to those of an oscillatory movement (frequency, velocity, and acceleration) [44]. Sound wave vibrations affect cell behavioural characteristics across orders of magnitude with lower and higher frequencies proposed to be frequency-dependent and constant, respectively [37]. In nature, sound vibrations from a buzzing bee were reported to induce sweeter nectar production in *Oenothera drummondii* flowers and the authors proposed this might lead to an increase in pollination [45]. Infra (low) and ultra (high) sonic frequencies can repel whiteflies (300 Hz) and moths (10 kHz) from tomato and strawberry crops, respectively [46, 47]. The right balance of bioacoustics can provide benefits for plant cell culture, postharvest fruit storage, and crop production systems [42, 43, 48]. Therefore, we herein interrogated the power-law behaviour of floral cells by examining the relationship between the frequency of vibration and self-pollination success of flowers from Endeavour, Sweetelle, Paulanca, and Managua tomato varieties.

We found that the peak amplitude velocity of floral vibrations induced by sonication was highest at lower sonic frequencies, decreased at higher frequencies, and acceleration was almost similar at both higher and lower sonic frequencies (Fig. 3E and F). The vibration velocity of the flower at 40 Hz for the mechanical shaker arm was twice that of the vibrating wand (Fig. 3B). Vibrations at high frequencies (450–1000 Hz) were reported to release more pollen than low frequency vibrations (100–400 Hz) [21]. It has been shown however, that floral vibrations transmitted to the anthers by bumblebees at lower frequencies (240–405 Hz) had only a modest effect on pollen ejection [29]. It has also been found that larger bees capable of generating higher frequencies release more pollen compared with smaller bees [49], suggesting that there may be additional effects of pollinator-specific buzzing that affect pollen removal [50]. Perhaps, the success of pollen extraction requires vibratory forces produced by the bee and its mass collectively give rise to an axial-bending dynamical property of the poricidal anther within the correct resonance [19]. The variation in frequency may not be important to affect pollen emission [29–31]. In contrast, duration and peak velocity amplitude are positively associated with pollen ejection during artificial floral vibration [29]. Contrarily, another study found a positive relationship between frequency and pollen release when expressing amplitude as displacement and velocity. However, when acceleration is used as a proxy for buzz amplitude, the relationship between frequency and pollen release becomes negative [31]. No significant differences were observed between the pollination treatments regarding seed set in four tomato fruit varieties (Figs 4–6). It seems likely that pollen ejection is equally influenced by multiple cell vibration characteristics, regardless of frequency, and hence, one variable may not be sufficient to describe the energy and force transmitted to the anther and hence underpin the success of pollen release. Our findings revealed a frequency-independent effect on seed set and self-pollination efficiency whereby pollen expulsion from the poricidal cone remained constant from infra- to near ultra-sonic frequency levels.

**Fig 8:**
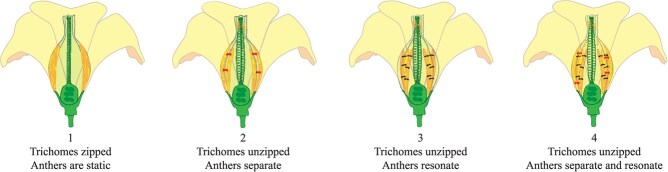
**Theoretical mechanisms associating trichome unzipping of the anther cone sheath with anther separation and/or resonance during sonic-induced tomato flower pollination.** Diagrammatic representation of a tomato flower depicting two anthers either side of the anther cone grove intertwined by trichomes (resembling a zipper) displayed in front of the stigma and style. (1) Untreated flower showing trichomes in a zipped state, where the anthers are static, fused, and cannot resonate. The unzipping of trichomes by sonication could cause the anthers to separate (2), resonate, (3) or both separate and resonate (4) leading to pollen ejection. Arrows indicate anther separation (red) and anther resonance (black). Pollen grains are depicted as orange dots (orange), anthers are elongated shapes with an outline, and trichomes are depicted as a zipper (e.g. clasp locker).

Mechanical pollination using a vibrating wand has been shown to increase tomato weight by 35.9% compared with an untreated control, while the nonbuzzing honeybee (*A. mellifera*) improved pollination and yield by only 28.3% [51]. We showed that lower sonication frequencies (50 Hz), the mechanical shaker arm, and vibrating wand were all sufficient to increase Sweetelle and Endeavour fruit weight by 94% to 100%; 71% to 86%. A near ultra-sonic frequency of 10 kHz also enhanced Sweetelle, Paulanca, Managua fruit weight to the same extent as the vibrating wand, yet boosted Endeavour fruit weight by 188%. Empirical studies showed that *Bombus huntii*–mediated pollination significantly enhanced fruit weight of two indeterminate tomato varieties Favorita and Sungold [52], and increased fruit production by bumblebee pollination in a series of determinate and indeterminate tomato varieties [53]. The greatest reported increase in fruit weight (74.5%) compared with an untreated control was attributed to *B. terrestris* that can floral buzz at 250–300 Hz frequency [51]. Similarly, *B. terrestris*–mediated pollination was reported to significantly increase marketable fruit quality when compared with mechanical vibration and auxin treatments [54, 55]. The energy transmitted from a pollinator to the floral structure could result from the combined interactive effect of both noncontact acoustic and contact-based mechanical cell vibrations [33]. The vibrations generated by the bee thoracic muscles are transmitted to the flower via direct physical contact with the anther [56, 57]. Our findings support this notion as the electric vibrating wand and mechanical shaking arm both operate by physically contacting the plant to induce floral vibrations. However, bioacoustics effectively triggered self-pollination and enlarged fruit size in four commercially cultivated tomato varieties without physical contact. Therefore, noncontact low frequency-dependent sonication movement of air particles, reminiscent of the vibrations produced by a buzzing bee, can also contribute to self-pollination and effectively boost fruit size.

Sonication and vibrating wand-induced cell vibrations revealed differences in fruit size (fruit height and width) and weight between the varieties (Figs 4–6). Higher sonic frequencies of 10 kHz boosted the size and weight of Endeavour but not Sweetelle, Paulanca, and Managua tomato fruits when compared to 50 Hz or the vibrating wand. The high frequency-dependent power-law cell behaviour exhibited by Endeavour flowers exposed to 10-kHz sonication could implicate an altered (epi)genetic basis to the boost in fruit size. One theory is that increasing frequency-dependent vibrations to floral cell gametes triggers single parent heterosis to enhance fruit vigour and boost tomato fruit size and weight (Fig. 4). This could resemble cross-pollination induced hybrid vigour commonly observed in the offspring from two genetically distinct parents. Plant breeding heterosis is widely used to boost crop vigour, enhance growth, flowering, seed set, and fruit size [58]. An alternative theory is that that increasing frequency-dependent vibrations affected the modulation of phytohormones that control gynoecium development. Ovule number and gynoecium size are usually correlated and upon fertilization the ovules develop into seeds while the gynoecium turns into a fruit [59]. These theories are plausible because sonication can alter gene expression in Arabidopsis and the epigenetic modulation of tomato fruit ripening [48, 60]. The enhanced size of Endeavour fruits exposed to increasing sonication frequency highlights that acoustic and substrate-borne components such as from a buzzing bee could both be essential to boost crop vigour, fruit size, and overall yield.

The mechanism by which pollen ejection is induced by vibrations has remained unclear. Our examination of buzz-pollinated anther cone sheath structures showed that sonic vibrations and the vibrating wand unentwined the interlocking trichomes and unravelled their meshed network that holds the lobes of anther cone sheath tightly in a zipped state (Fig. 8). There are trichome hairs internally within the anther cone sheath that might also contribute as structural features to control pollen dispersal. So, what role might the anther trichomes play in cleistogamy? One theory is that the separation of trichomes could transfer energy to assist the separation of the anthers and thereby increase their elasticity to open the cone walls and facilitate pollen expulsion. A second theory is that sonication induces anther resonance during the unzipping of trichomes and yet does not induce anther separation. A third theory is that trichome unzipping facilitates both anther separation and anther resonance (Fig. 8). It is thought that the acceleration of the anther stylar tip during floral vibrations generates centrifugal forces on the pollen grains causing them to be expelled from the apical pore [30]. Elastic collisions against the internal walls of the vibrating anther are also thought to transfer kinetic energy to the pollen grains, which causes expulsion [36, 61]. As the energy increases, the particles begin to move and escape through the apical pore. Pollen release is a function of anther geometry, velocity and the frequency of the vibrations transmitted from the bee or artificial pollination to the anthers. The mechanical effects and electrostatic interactions between the pollen grains and the anther walls can play a significant role in expelling pollen from anthers [61]. The combined effects of centrifugal forces, kinetic energy and electrostatic interactions generated by floral vibrations likely contribute to the expulsion of pollen grains. A low-power frequency dependent cell vibration like that emitted by a buzzing bee could potentially unzip their meshed trichome network perhaps required for cleistogamy and achieve a considerably higher fruit set following self-pollination.

Modern technology and advanced research methods that automate precision self-pollination devices would be particularly valuable for the protected cropping industry and in particular high-tech glasshouses, where buzzing bees are not suitable for the environment [10]. Importation of commercially reared bumblebees is not permitted in countries like Australia due to their potential in becoming an agronomic pest and threat to the ecological environment [7]. Handheld mechanical pollination devices are the best suitable alternative to natural bee pollination for tomatoes grown in protected cropping environments; however, labour costs can be prohibitive to large-scale industry applications [11]. Contactless precision sonication technology that simulates a buzzing bee can be automated to promote effective self-pollination, reduce labour costs, and depending upon the variety potentially enlarge fruit size to boost overall crop production.

## Materials and methods

### Plant material

Seeds from four commercial tomato cultivars were sourced: Sweetelle from Syngenta (Australia) and Endeavour, Paulanca, and Managua from Rijk Zwaan (Australia), through Perfection Fresh (Australia Pty Ltd) Homebush, Australia. Seeds were sown in Seed and Cutting Premium Potting Mix (Scotts Osmocote) in 30-cell plastic trays covered with transparent film to maintain an optimum germination temperature of 27°C and humidity. At the two-leaf stage, seedlings were transplanted to 50 L pots (10 pots per variety) containing Tomato, Vegetable & Herb Premium Potting Mix (Scotts Osmocote) and grown in a glasshouse located at Western Sydney University in Richmond, New South Wales (33.6000^o^S, 150.7500°E). Plants were maintained at 25°C during the day and 18°C at night. Plants were supplied with a commercial granular fertilizer (Aquasol; NPK 23: 3.95: 14) every 2 weeks and watered every other day to maintain soil moisture. Secondary shoots and trusses were pruned weekly as per best horticultural practices [41].

### Vibrational frequency quantification using a laser vibrometer

To capture plant vibrational velocity responses to mechanical and sonic vibrations, we utilized a helium-neon laser head coupled with a PSV-500 scanning vibrometer (Polytech). The desired frequency range was generated between 50 Hz to 10 kHz using a high-frequency signal generator connected to an amplifier and a data acquisition (DAQ) unit (Polytech). The vibrating wand (Vibri tomato pollinator Vario 12v) and mechanical shaker arm were placed on the stem below the truss to trigger floral vibrations. The sonic vibrations were induced by exciting a high-power subwoofer speaker placed 5 cm from the flower on a single truss (Fig. 1). Measurements were performed in triplicate per flower. Data were captured both in time and frequency domain using a Fast Fourier Transform (FFT) algorithm. The equivalent FFT is plotted for the time series data to compare the vibrational signals (velocity in mm/s) within the frequency domain. The frequency values corresponded to the highest velocity signals identified from floral vibrations. Data were stored and processed using the DAQ and DIAdem NI software, respectively. The intensity test was conducted to find the sound pressure level of the subwoofer using a decibel meter (Decibel-X pro sound meter).

### Floral pollination treatments

Control plants were grown in a separate glasshouse bay to reduce cross-contamination from sonic stimulation events and determine background self-pollination efficacy. Airflow was minimized and plants were spaced to avoid direct contact with the air venting system. For sonic treatments, a signal generator, and a linear digital switching (LDS) amplifier without attenuator connected to a 10 cm diameter subwoofer speaker (Vibe Blackair 1300 Watts and root mean square (RMS) power of 450 Watts) had a frequency response of 30 to 30 000 Hz and a sensitivity of 88 dB. Prior to any vibrational stimulus, flowers from Endeavour and Sweetelle trusses were trimmed leaving only four to six mature flowers. Flowers from various trusses of the Endeavour cultivar were exposed to single sonic frequencies of 50, 180, 250, 900, and 10 000 Hz for 5 minutes. Flowers from various trusses of the Sweetelle cultivar were exposed to frequencies of 250 and 10 000 Hz for 5 minutes. Flowers from Paulunca and Managua truss 1 and 2 were sequentially upon maturity exposed to sonic frequencies of 10 000 Hz for 5 minutes. Artificial floral vibrations using a vibrating wand (40 Hz; Vibri tomato pollinator Vario 12v) or an aluminium rod arm attached to a permanent magnet shaker unit mounted on a strong metal frame (operated within a 40- to 1000-Hz range when connected to the LDS amplifier) were performed for 5 seconds by stimulating the plant stem directly below the truss. A Charge Accelerometer (4393), charge amplifier, and DAQ (DIAdem NI software) were used to identify the optimal frequency of the electric vibrating probe. Frequency and acceleration responses of the flower were assessed using a laser vibrometer within the glasshouse bays.

### Postharvest traits

The efficacy of pollination treatments was assessed by counting the number of seeds per fruit, total seed weight per fruit (mg), fruit height (cm), fruit width (cm), and fruit weight (g) at the mature ripe stage of horticultural maturity.

### Scanning electron microscopy of tomato flowers

Tomato flowers were dehydrated progressing through an ethanol series of 70%, 90%, and 100% for 30 minutes. Dehydrated samples were then critical-point dried (Leica EM CPD300), mounted onto scanning electron microscopy (SEM) stubs using double-sided conductive carbon tabs, and gold coated using a Leica EM SCF SCD005 cool sputtering device. Samples were examined using a Hitachi FlexSEM 1000II SEM under high vacuum at an accelerating voltage of 5 kV (surface observation), spot intensity of 40 and working distance of 5 mm. The anther cone sheath is divided into three sections: the upper anther sheath, middle sheath, and lower sheath. Images were collected from this section using a secondary imagery detector and microanalysis energy dispersive spectroscopy detector (Bruker Espisrit EDS). Separated trichomes are those that do not share their outer boundary with any other trichomes. Partially separated trichomes are those that share their outer boundary with one or two other trichomes. Unseparated trichomes are those that cannot be individually counted due to the lack of clear outer boundaries.

### Statistical analysis

Statistical significance was calculated by performing a one-way analysis of variance (ANOVA) followed by a *post hoc* Tukey’s test using Sigma Plot 14.0 software. There were 10 models, where five fruit traits were analysed across different frequency-dependent vibrational response mechanisms relative to their untreated controls, and we analysed each cultivar separately.

## Data Availability

All data are available in the main text or the supplementary materials. The data that support the findings of this study are available from the corresponding author upon reasonable request.
